# Comparison of visual estimation and quantitative measurement of left ventricular ejection fraction in untrained perioperative echocardiographers

**DOI:** 10.1186/s12871-023-02067-3

**Published:** 2023-04-01

**Authors:** Kasana Raksamani, Apinya Noirit, Nophanan Chaikittisilpa

**Affiliations:** Department of Anesthesiology, Faculty of Medicine Siriraj Hospital, 2 Wanglang Road, Bangkok Noi, Bangkok, Thailand

**Keywords:** Visual estimation, Eyeballing, Ventricular ejection fraction, Rescue echocardiography, Perioperative echocardiography, Untrained

## Abstract

**Background:**

Perioperative evaluation of the left ventricular systolic function is essential information to help diagnose and manage life-threatening perioperative emergencies. Although quantifying the left ventricular ejection fraction (LVEF) is recommended to determine the left ventricular function, it may not always be feasible in emergency perioperative settings. This study compared the visual estimation of LVEF (eyeballing) by noncardiac anesthesiologists with the quantitative LVEF measured using a modified Simpson’s biplane method.

**Methods:**

Transesophageal echocardiographic (TEE) studies of 35 patients were selected and 3 different echocardiographic views (the mid-esophageal four chamber view, the mid-esophageal two chamber view, and the transgastric mid-papillary short axis view) were recovered from each study and displayed in random order. Two cardiac anesthesiologists certified in perioperative echocardiography independently measured LVEF using the modified Simpson method and categorized LVEF into five grades: hyperdynamic LVEF, normal, mildly reduced LVEF, moderately reduced LVEF and severely reduced LVEF. Seven noncardiac anesthesiologists with limited experience in echocardiography also reviewed the same TEE studies and estimated the LVEF and graded LV function. The precision of the LV function classification and the correlation between visual estimation of LVEF and quantitative LVEF were calculated. The agreement of measurements between the two methods was also assessed.

**Results:**

Pearson’s correlation between the LVEF estimated by the participants and the quantitative LVEF using the modified Simpson method was 0.818 (*p* < 0.001). Of a total of 245 responses, 120 (49.0%) responses were correct grading of the LV function. Participants were able to classify the LV function more accurately in the LV function grades 1 and 5 (65.3%). The 95% level of agreement of the Bland-Altman method was − 11.3-24.5. -21.9-22.6, − 23.1-26.5, − 20.5-22.0 and − 26.6-11.1 for LV grade 1 to 5, respectively.

**Conclusion:**

Visual estimation of LVEF in perioperative TEE has acceptable accuracy in untrained echocardiographers and can be used for rescue TEE.

## Highlights

• Estimation of LVEF by noncardiac anesthesiologists is essential to aid in diagnosing and managing life-threatening perioperative conditions.

• Visual estimation of the LVEF is easy to perform with acceptable accuracy.

• This method could be helpful as part of a rescue TEE protocol and in training programs for LVEF interpretation.

## Introduction

Transesophageal echocardiography (TEE) is used for hemodynamic monitoring and to provide immediate assessment of cardiac function for anesthesiologists during cardiac surgery [1–3]. Increasingly, TEE has become a useful clinical tool during noncardiac surgery in high-risk patients, as well as for therapeutic decision making for emergency resuscitation [4, 5]. The recommended guidelines for the rescue of TEE are simple and straightforward, making it possible for non-expert providers to perform in acutely unstable patients [5, 6].

The left ventricular ejection fraction (LVEF) is a widely used and accepted key parameter for assessing left ventricular (LV) function. Although calculating the two-dimensional (2D) LVEF using the modified Simpson method of discs is preferred [7–9], this method requires optimal imaging conditions and extensive practice to achieve a high level of precision when evaluating LVEF [7]. LVEF is frequently rapidly evaluated with the visual estimation technique [5, 10], particularly during emergency conditions. Visual estimation of LVEF by experienced echocardiographers has been reported to be strongly correlated with the modified Simpson method [11, 12]. However, its precision has not been determined in untrained echocardiographers. During emergency perioperative settings, experienced echocardiographers may not always be available, and attending anesthesiologists with limited experience in echocardiography may need to perform rescue TEE and assess LV function using estimated LVEF [6, 10]. Furthermore, measuring the LVEF using modified Simpson technique is challenging to perform in time-sensitive circumstances [5]. Therefore, an accurate estimate of LVEF by noncardiac anesthesiologists is essential to aid the diagnosis and management of life-threatening perioperative conditions.

Previous studies evaluating the precision of visual estimation of LVEF using transthoracic echocardiography (TTE) imaging views demonstrated 66–72% agreement between visual LVEF and quantitative methods among noncardiologists (intensivists, emergency physicians, and medical students) with minimal training [13–15]. We hypothesized that visual estimation of the LVEF should also be a feasible method for noncardiac anesthesiologists using imaging views from TEE.

Therefore, the study aimed to find the agreement on the LV function classification between the visual estimation of noncardiac anesthesiologists and the reference method of quantitative measurement using modified Simpson biplane discs.

## Methods

This study was approved by the Siriraj Institutional Review Board (Si 201/2020) and written informed consent was obtained from all participants.

### Echocardiographic studies

The TEE imaging studies were evaluated for image quality and 40 sets of intraoperative TEE studies were selected from the image management system database of the Department of Anesthesiology, Siriraj Hospital Faculty of Medicine, Bangkok (Xcelera V1.2 L4 SP2, Philips, Amsterdam, The Netherlands) by two researchers (AN and KR). The TEE imaging studies were anonymously retrieved and three video clips were exported for each study set, including the mid-esophageal four chamber view, the mid-esophageal two chamber view, and the transgastric mid-papillary short axis view [4, 5]. Two cardiac anesthesiologists (KR and NC) with perioperative echocardiography certification independently measured LVEF using the modified Simpson method and categorized the LVEF into five categories according to the recommendation of the American College of Cardiology: grade 1 hyperdynamic LV (LVEF > 70%), grade 2 normal LV (LVEF 50–69.9%), grade 3 mildly reduced LVEF (LVEF 40–49.9%), grade 4 moderately reduced LVEF (LVEF 30–39.9%), and grade 5 severely reduced LVEF (EF < 30%) [16]. Finally, seven files per each LV function category (35 total) were selected. All TEE imaging files were exported and inserted into a PowerPoint slideshow in random order (randomization.org).

### Participants

For the purpose of the study, seven noncardiac anesthesiologists with limited experience in echocardiography (no previous training in perioperative TTE/ TEE) were recruited using purposeful sampling. All participants received a 10-minute reading on the concept of LVEF measurement, the orientation and planes of the images, the structures demonstrated in each view, and the LVEF classifications. The participants were then asked to review the same 35 sets of TEE imaging studies, estimate LVEF, and grade the LV function category without time limitation. All participants were blinded to the LVEF and the grade of the LV function measured by experts. Participants received immediate feedback on their responses compared to the quantitative LVEF and their grading after each set of clips once their responses were submitted. Age, sex, and experience in echocardiography of the participants were recorded. The participants were then asked to rate their confidence in the accuracy of their estimates after completion.

### Statistical analysis

The primary outcome of this study was the agreement of the LV function classification between the estimated LVEF of participants and the quantitative LVEF of trained experts. Acceptable agreement was determined when the participant and expert grades matched or were in adjacent categories (1 grading difference). The secondary outcome was the correlation between the LVEF estimated by the participants and the quantitative LVEF. The agreement between each LVEF estimated by the participants and the quantitative LVEF using the modified Simpson method was also evaluated.

The sample size calculation was informed by a previous study that reported that emergency physicians and cardiologists agreed that the systolic function classification of LV was 0.9 [13]. Based on the confidence interval for proportional studies using nQuery Advisor 6.0., TEE imaging studies of 35 patients would give a 95% confidence level to determine the accuracy of the LV function classification.

Statistical analyses were performed using SPSS 21 for Mac OS (Chicago, USA). Descriptive statistics are presented as number (percentage), mean (standard deviation), and median (interquartile range), where appropriate. Correlation was assessed using scatter plots and Pearson’s correlation coefficients. A Bland-Altman scheme was used to demonstrate agreement of LVEF between the estimates of the participants and quantitative LVEF. The bias and 95% limits of agreement (LOA) were calculated. A *p*-value < 0.05 was considered statistically significant.

## Results

From the 35 TEE imaging studies, the mean LVEF measured using the modified Simpson method was 45.3% ± 19.8%, and the mean LVEF in each grade from 1 to 5 was 74.0% ± 0.8, 56.7% ± 1.9, 44.6% ± 0.6, 33.2% ± 0.5, and 17.8% ± 2.1%, respectively. The interrater reliability of the quantitative LVEF between the two authors was 0.97.

Seven noncardiac anesthesiologists with a mean age of 38.1 ± 8.2 years and a median experience (IQR) of seven (4,13) years were recruited. A total of 245 responses from seven participants were collected and 120 (49%) of the visual estimation of LV function gradings were correct. For the LV function classifications, 32 (65.3%) of the answers in both extreme gradings (Grade 1: hyperdynamics and grade 5: severely reduced LVEF) were the same as those classified by the modified Simpson method. Meanwhile, only 29 (59.2%), 13 (26.5%) and 14 (28.6%) responses were correct in Grade 2 (normal LVEF), Grade 3 (mildly reduced LVEF), and Grade 4 (moderately reduced LVEF), respectively. For estimates that did not match the LV function categories in the quantitative LVEF, 108 (44.1%) were in adjacent categories (1 grading difference), 15 (6.1%) answers were unmatched by two grading categories, and only two answers (0.8%) were unmatched by more than two gradings (Table 1).

**Table 1:**
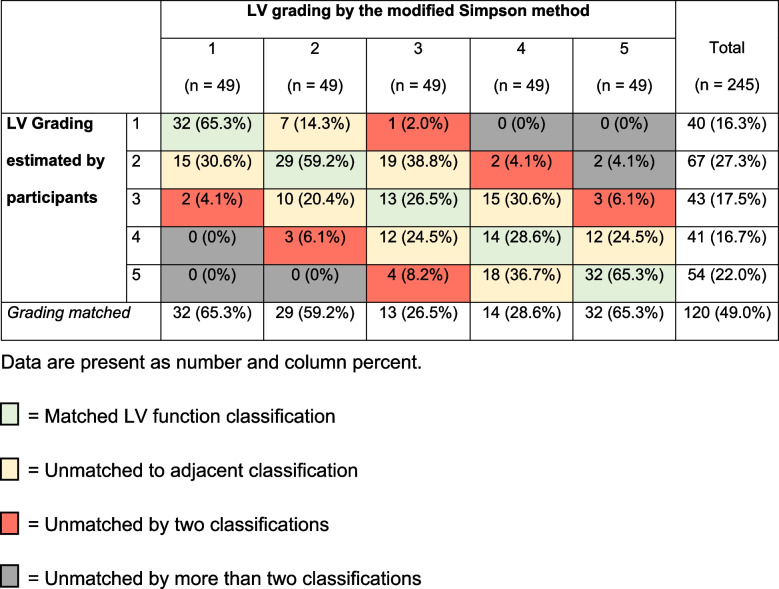
Visual estimation of LV function classifications by participants and modified Simpson’s method

**Fig. 1:**
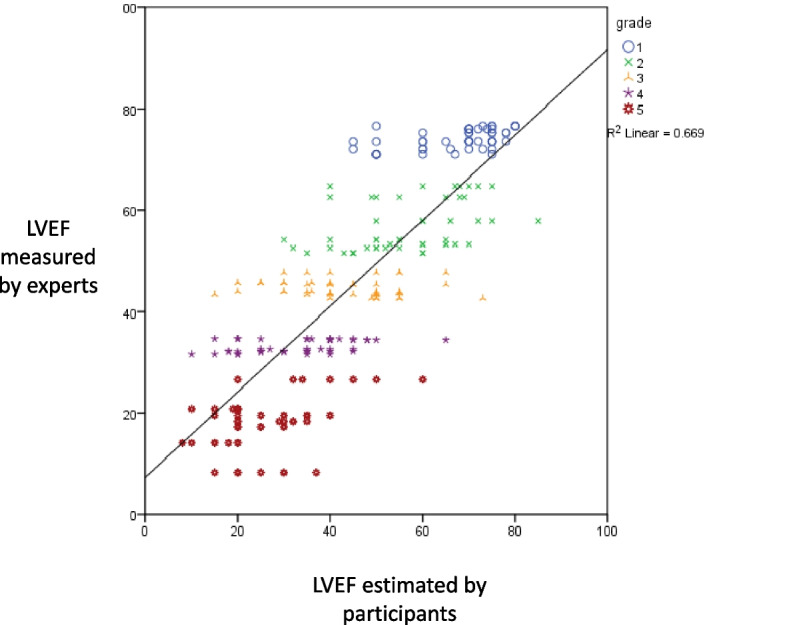
Scatter plot demonstrating the correlation between LVEF estimated by participants and measured by experts. *n* = 245, r^2^ = 0.669

**Fig. 2:**
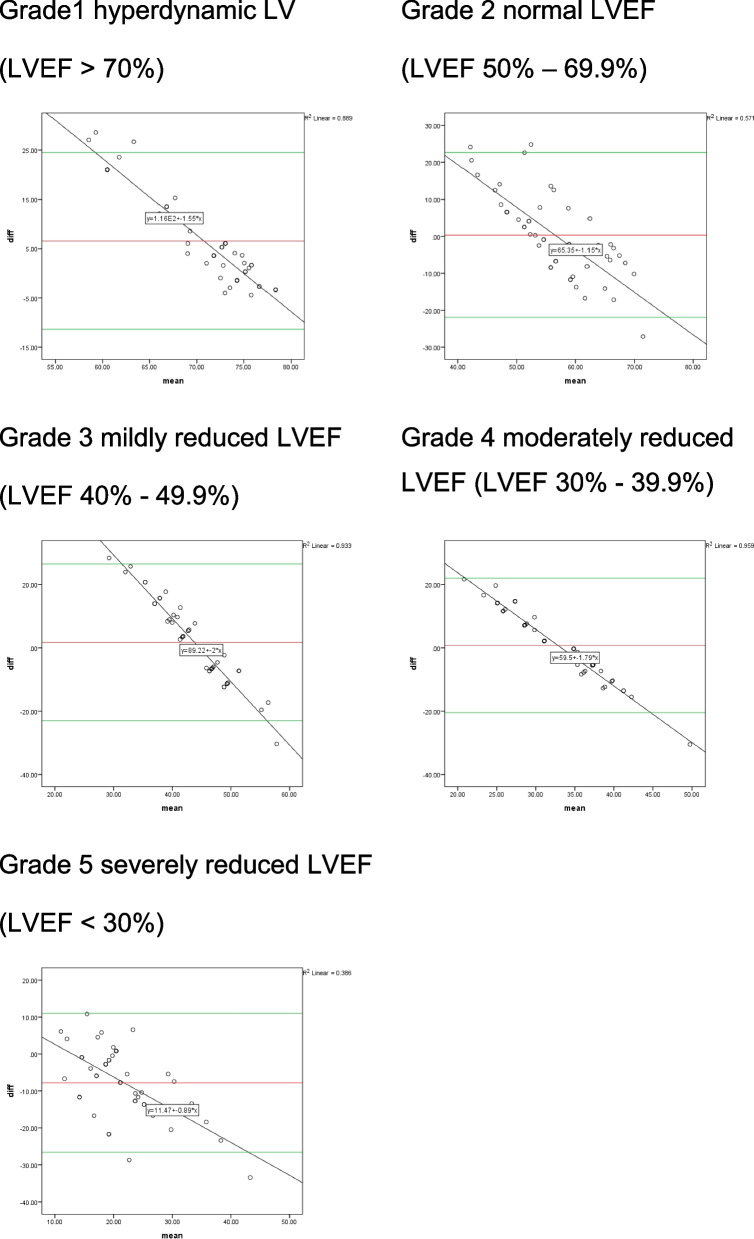
Bland-Altman agreement plots demonstrate the difference between LVEF estimated by participants and experts for each grade with a logarithmic horizontal scale. The X-axis represented the mean of the LVEF estimated by participants and experts for each grade. The Y-axis represented the difference between LVEF estimated by participants and experts for each grade. The red line indicates mean LVEF differences (%), and the green line represents the 95% limits of agreement

The interrater reliability of the seven noncardiac anesthesiologists who estimated LVEF had an intraclass correlation (ICC) of 0.961 [95% confidence interval (CI), 0.935 to 0.978]. The ICC was 0.754 (95% CI 0.366–0.949), 0.811 (95% CI 0.508–0.961), 0.769 (95% CI 0.417–0.952), 0.876 (95% CI 0.669–0.975), and 0.881 (95% CI 0.681–0.976) for the grade 1 to 5 respectively. The scatter plot shows a moderate, positive, linear association between the estimated LVEF by the participants and the quantified LVEF using the modified Simpson method (Fig. 1). While Pearson’s correlation demonstrated a strong correlation between these two variables (R = 0.818, *p* < 0.001), the Bland-Altman plot method revealed no systematic errors, and the plot for each LV function classification was in the same distribution pattern. The 95% LOA of the Bland-Altman method ranged from − 11.3 to 24.5, -21.9 to 22.6, − 23.1 to 26.5, − 20.5 to 22.0 and − 26.6 to 11.1 for LV grade 1 to 5, respectively (Fig. 2).

## Discussion

### Summary of the findings

The findings of this study suggest a fair correlation between visual LVEF estimated by noncardiac anesthesiologists without experience in echocardiographic training and quantified LVEF using the modified Simpson method. Although only 40% of the participants were able to correctly grade the LV function, most of the unmatched responses were in the adjacent categories (misplaced by one grading category).

Visual estimation of LVEF is accurate and reliable in trained and experienced echocardiographers [11, 12]. Our objective was to determine whether this method is also precise in untrained echocardiographers. We observed that untrained echocardiographers accurately graded the LV function as high as 65% in extreme classifications (hyperdynamic LV and severely reduced LVEF). There was a strong correlation between the LVEF estimated by the untrained echocardiographers and that measured by experts.

### Estimated assessment of LVEF

Both the 2D and 3D volumetric methods accurately quantify LVEF better than the linear dimension method [7–9]. The most popular method for 2D echocardiography is the modified Simpson biplane method of disks. However, this method takes time to measure and may not be suitable for unpredictable/ fast-changing perioperative settings [17]. In addition to LVEF measurement, the American College of Cardiology recommended grading of left ventricular function to interpret left ventricular function [16]. The classification of LV function ranged from hyperdynamic LV to severely reduced LV function, making it practical for the clinician to analyze LV function and diagnose clinical conditions. Cole et al. studied the classification of left ventricular function using the visual estimation technique by making the echocardiographers classify each clip twice. They reported the repeated grading reliability of the same operator to be 68% [18]. The reliability rate was not high due to the mixed level of experience in echocardiography; only half of the operators were certified echocardiographers.

Furthermore, based on our clinical experience, the difference in a single grade may not impact perioperative hemodynamic management. When combining the correct classification and ± 1 quality different from the correct classification, we found that untrained echocardiographers classified LV function within clinically acceptable ranges in 93.1% of cases. When applying this to rescue echocardiography, only the extreme gradation of hyperdynamic and severely reduced LV was an important cause of unstable patients in the perioperative period [19, 20]. The middle categories are not entirely helpful in providing information on rescue echocardiography [6]. This study revealed the correct grade of the participants of 65.3% in both extreme grades.

### Clinical implication

The visual method of estimating LVEF has been closely correlated with other methods of assessing LVEF [11–13, 21]. Most studies correlated LVEF between visual estimation and quantifying methods in experienced echocardiographers [11, 12, 21]. Shahgaldi et al. reported an R of 0.91 for 2D and 0.95 for triplane echocardiography estimated by experienced echocardiographers and quantitative real-time three-dimensional echocardiography [12]. However, Unluer et al. studied this estimation technique in novice emergency physicians after a brief training program and demonstrated a strong correlation with the LVEF measured by cardiologists [13]. We showed that the LVEF estimated by untrained echocardiographers is highly correlated (r = 0.818) with the LVEF measured by experts. This may be due to the immediate feedback participants received during the review process in this study. Akinboboye et al. proposed that an acceptable learning curve of visual estimation of LVEF occurred after 20 cases had been reviewed with instant feedback [22]. In comparison, Lee et al. reported a minimum of 55 case reviews with feedback to effectively train LVEF visual estimation in medical students [23]. This study used 35 cases with instant feedback because our objective was to evaluate the ability to estimate the LVEF rather than training. The ease of the visual LVEF estimation learning process suggests that it could be helpful in training novice echocardiographers to interpret LV function in emergency situations.

One of the most important parameters needed to manage hemodynamic problems is the systolic function of the LV [19]. Echocardiography is increasingly used to diagnose life-threatening hemodynamic problems, known as ‘rescue echocardiography’ [4, 5, 19, 24]. Visual estimation of LV function is ideally suited for a high-volume, point-of-care echocardiography program due to its simplicity. Our study confirmed that visual evaluation of LV function provides a good LV function grading and strongly correlates with the LVEF measured by experts.

This study has limitations. The nonexperimental nature of the study could not control many factors, such as the time the participants used for the study and their familiarity with the ultrasound images of the participants. Further studies with a more rigorous design would be valuable. In addition, this study focused only on the visual estimation of the LVEF. It did not include other skills and measurements to help diagnose during rescue TEE, such as probe placement, knobology, and image profiles to help diagnose specific conditions such as cardiac tamponade and embolic phenomena.

## Conclusions

Visual estimation of LVEF is easy to perform with acceptable accuracy in untrained echocardiographers with minimal training. This method could be helpful as part of a rescue TEE protocol and in training programs for LVEF interpretation.

## Data Availability

The datasets used and/or analyzed during the current study are available from the corresponding author on reasonable request.
